# ETV4: an emerging target in pancreatic cancer

**DOI:** 10.18632/oncoscience.471

**Published:** 2018-10-11

**Authors:** Sachin Kumar Deshmukh, Ajay P. Singh, Seema Singh

**Affiliations:** Mitchell Cancer Institute, University of South Alabama, Mobile, AL 36604-1405, USA

**Keywords:** pancreatic cancer, ETV4, cyclin D1

Recently, pancreatic cancer (PC) became the third leading cause of cancer-related death in the United States, which is expected to inflict approximately 55,440 Americans in 2018 and take 44,330 lives [1]. This emphasizes the need to develop novel strategies to curtail the increasing incidence and death rates of PC, which could only be possible by making progress on several fronts. First, we need to identify novel diagnostic targets and develop assays, so that pancreatic cancer can be detected early at a more manageable stage. Second, we need to understand the biology and molecular mechanisms that drive the rapid and aggressive progression, and drug- resistant nature of this malignancy. Third, we need to identify functional targets that can be useful for prognostic assessment and treatment planning. Lastly, we need to develop novel therapeutic approaches that are effective and overcome the limitations associated with current therapy.

Years of molecular research have identified several genetic and epigenetic aberrations and many of which have been confirmed to play important roles in pathogenic processes [2]. Since PC is one of the most genetically advanced malignancies at the time of diagnosis, it is important that we continue our efforts to find and characterize novel gene targets of pathological significance, while looking for novel ways to clinical exploit currently established targets. In that line, we recently investigated the functional significance of E26 transformation-specific (ETS) variant 4 (ETV4) in PC. ETV4, also known as PEA3 and E1AF, is a member of the polyomavirus enhancer activator 3 (PEA3) subfamily of ETS-oncogene transcription factors that play a crucial role in both normal physiology and pathological conditions [3]. Using clinical specimens of normal and malignant pancreatic tissues and established cancer cell lines, we demonstrated an aberrant expression of ETV4 in PC. Furthermore, we also experimentally established an association of ETV4 overexpression with enhanced growth and rapid cell-cycle progression of PC cells. Silencing of ETV4 in two PC cell lines (ASPC1 and Colo357) reduced their growth, while its forced overexpression in another cell line (BXPC3) led to an increase in the growth, compared to their respective control cells. ETV4- induced cell growth resulted from the rapid transition of cells from G1- to S-phase of the cell cycle due to an increased expression of *Cyclin D1 (CCND1)*, a protein crucial for cell-cycle progression from G1- to S-phase. ETV4 transcriptionally upregulated *CCND1* expression by direct binding to its promoter, and effects of ETV4 silencing could be reversed by the forced expression of CCND1 in PC cells [4].

**Figure 1:**
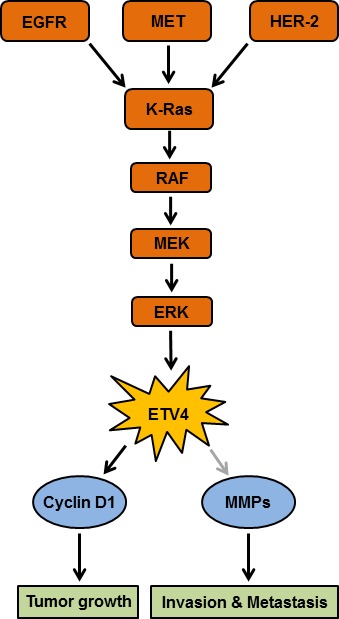
Proposed model for ETV4 (E26 transformation-specific (ETS) variant 4) overexpression that is involved in the pathobiology of pancreatic cancer ETV4 overexpression can be mediated by multiple signaling events including activation of K-Ras. Binding of a ligand specific to EGFR and MET receptors leads to activation of K-Ras which activates RAF, mitogen-activated protein kinase (MEK) and extracellular signal-regulated kinase (ERK) signaling pathways. These signaling events subsequently activate transcription factor ETV4. The activated ETV4 upregulates the expression of cyclin D1 and MMPs (matrix metalloproteinase) leading to tumor growth, invasion, and metastasis. Gray arrow indicates that the connection of signaling molecules with ETV4 recognized in other malignancies, however, it is still unexplored in pancreatic cancer.

Overexpression and pathologic involvement of ETV4 has been reported in a variety of other tumors as well including prostate, breast, lung, colon, etc. [3]. A correlation of ETV4 with HER2/Neu overexpression, tumor grade, and recurrence in human breast cancer patients has also been reported [5]. ETV4 modulates cell cycle regulation and Wnt/β-catenin signaling, which is important in potentiating gastrointestinal stromal tumor malignancy. Knockdown of ETV4 suppressed tumor cell proliferation, growth, and aggressiveness of gastrointestinal stromal tumor [6]. ETV4 is also shown to regulate MYC, and other genes responsible for proliferation in prostate cancer cells and overexpression of ETV4 leads to invasion and metastatic potential by epithelial-to-mesenchymall transition, whereas inhibition of ETV4 retarded the proliferation and invasion of prostate cancer cells [7]. Of particular significance to PC, a connection of ETV4 with other established molecular targets of relevance such as K-Ras and MET signaling has been reported in different malignancies [8]. Validation of these findings in PC may place ETV4 on the list of key pathological drivers of pancreatic carcinogenesis. However, to exploit ETV4 as a therapeutic target would demand a deep biochemical understanding of ETV4 and its interactions with other proteins, and of the mechanisms underlying its aberrant expression in PC (Figure 1). Hence, our recent findings although highly significant are just the beginning of the unfolding of the potential multi-faceted function of ETV4 in PC and future investigations would pave the way for its clinical exploitation.
